# Supplementary material for: Sequence learning recodes cortical representations instead of strengthening initial ones
Source: PLoS Comput Biol. 2021 May 24;17(5):e1008969. doi: 10.1371/journal.pcbi.1008969 (PMC8177667; doi:10.1371/journal.pcbi.1008969)

S1 Text. Neural representation of novel sequences

To identify which brain regions were involved in novel sequence representation we formally defined two classical sequence representation models: item-position associations, where sequences are formed by mapping items to their ordinal positions; and item-item associations, where consecutive items are associated with each other. We also defined an additional control model which tested for a null-hypothesis that neural activity reflects a superposition of item representations instead, called the *item mixture model*.

We only considered evidence for a sequence representation model to be significant in a brain region if it satisfied three criteria: (1) the model correlation with data was significantly greater across participants than the lower bound of the noise ceiling, (2) the lower bound of the noise ceiling was significantly greater than zero across participants, and (3) the average correlation for the item mixture model (null-hypothesis) did not reach the noise ceiling in that region.

The noise ceiling gives theoretical lower and upper bounds of the possible model fit given the noise in the data: any representational model which does not reach the noise ceiling should not be considered as a plausible explanation of voxel responses (see Noise ceiling estimation in Methods). We found evidence for novel sequence representation in eleven regions in the dorsal visual processing stream (Fig A).

Fig A: Novel sequence representation in the dorsal visual processing stream. The bar plots show how well the associative item-position representation model predicted the distance between pairs of voxel activity patterns corresponding to individual novel sequences. Y-axis displays the model evidence in terms of participants' average Spearman's rank-order correlation r, error bars represent the standard error of the mean (SEM); dashed lines in bar plots represent the lower and upper bounds of the noise ceiling. X-axis shows the results separately for all three task phases (P - presentation, D - delay, R - response; Fig 2). Red asterisks mark regions and task phases where the correlation with the model prediction was significantly greater than the noise ceiling and compared to the item mixture model ($df = 21, p < 10^{-3}$), the item mixture model did not reach the noise ceiling, and the lower noise ceiling was significantly greater than zero across participants.

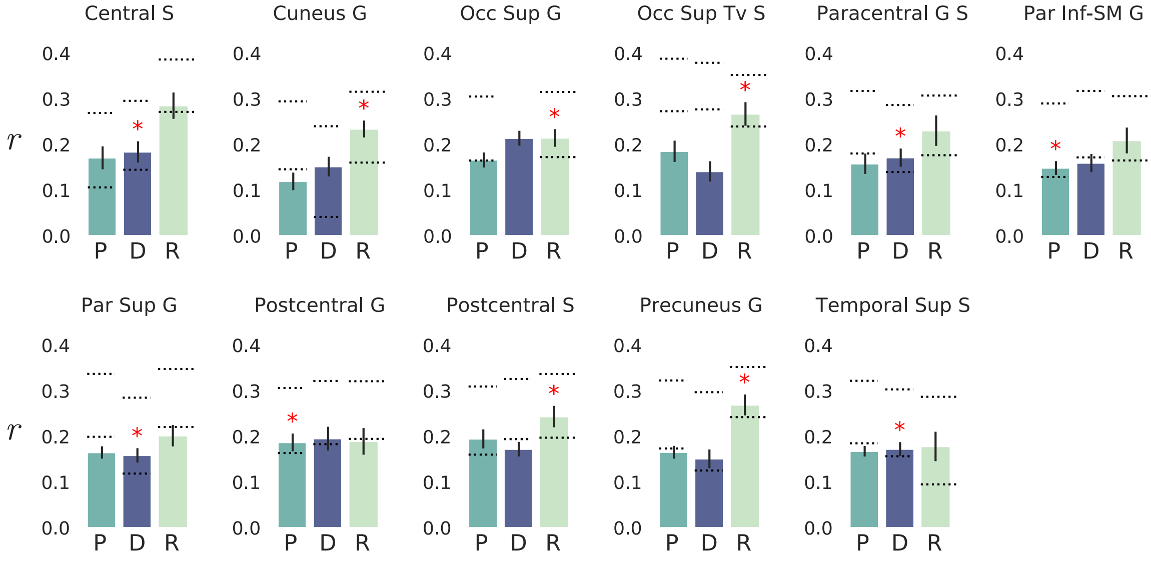

Supplement: S1 Text — (PDF) [file pcbi.1008969.s001.pdf]
