# Supplementary material for: Sequence learning recodes cortical representations instead of strengthening initial ones
Source: PLoS Comput Biol. 2021 May 24;17(5):e1008969. doi: 10.1371/journal.pcbi.1008969 (PMC8177667; doi:10.1371/journal.pcbi.1008969)
Supplement: S2 Text — (PDF) [file pcbi.1008969.s002.pdf]

S2 Text. Item mixture model parameters

There are a number of meaningful ways individual items could contribute to the mixture. Although we have chosen a 'recency mixture' where the most recent item contributes the most, we could have also used a 'primacy mixture' with exactly the opposite slope of mixture contributions. The reason we only tested for the 'recency mixture' is that both recency and primacy models predict the same similarity between individual sequences in our task. In other words, a representational dissimilarity matrix (RDM, Eq 21) derived with a recency-based item mixture predicts the same similarity between voxel patterns as an RDM derived with a primacy based mixture (if the absolute value of the gradient slope remains the same). For a detailed explanation see the example below.

We could have chosen any of the infinite slope values across positions. However we chose a middle point between two extreme slope values: all the mixtures become the same when the slope is horizontally flat and only a single item contributes when the slope is vertical. We could have obtained an estimate of the coefficients from analysing the individual finger movement representations since the mapping between items and fingers was randomised across the participants. However, here our focus was on sequence representations and therefore we felt a null hypothesis representing an average in the space of possible mixtures was enough.

Worked example

We assume a recency mixture model where contributions increase with sequence position as $\beta = [0, 1/6, 1/3, 1/2]$, then we can represent a sequence as an item mixture in our task by indicating the proportion of each four items $[A, B, C, D]$ in the mixture, e.g.: $[C, A, D, B]$ as $[A : 1/6, B : 1/2, C : 0, D : 1/3]$, $[B, D, C, A]$ as $[A : 1/3, B : 0, C : 1/6, D : 1/2]$ and so forth. Given that item representations do not change from sequence to sequence and hence all mixtures would be equal if the coefficients β were equal across all items (e.g. uniformly 1/4) the distances between the resulting mixture representations are determined by the vector of coefficients. For example, the euclidean distance between $[A, B, C, D]$ and $[C, A, D, B]$ as mixtures (given $\beta = [0, 1/6, 1/3, 1/2]$) is the euclidean distance between the two four-dimensional mixture vectors:

$d = EuclideanDist([1/6, 1/2, 0, 1/3], [1/3, 0, 1/6, 1/2])$

Assuming the gradient β has always the same number of unique values then the distance between such 4D points depends only on the absolute value of the gradient slope and not the direction of it (positive or negative slope). This should be evident when one considers that in our task all sequences have always exactly the same four items and hence mixture contributions are directly proportional to the ordering of the same four items.

For a simulation how the mixture model similarity prediction does not depend on the

direction of the slope (recency vs. primacy) see the Jupyter Notebook (*model_mixture*) at our code repository.
