# Supplementary material for: Sequence learning recodes cortical representations instead of strengthening initial ones
Source: PLoS Comput Biol. 2021 May 24;17(5):e1008969. doi: 10.1371/journal.pcbi.1008969 (PMC8177667; doi:10.1371/journal.pcbi.1008969)
Supplement: S3 Text — (PDF) [file pcbi.1008969.s003.pdf]

S3 Text. Optimal chunking model estimation

We want to estimate the posterior probability distribution of chunking models θ given the observed data D (presented sequences):

$$p(\theta|D) = \frac{p(D|\theta)p(\theta)}{p(D)}, \tag{1}$$

and choose the model with the highest posterior probability:

$$\theta^{MAP} = \underset{\theta}{\operatorname{argmax}}[p(\theta|D)].$$

Since Bayesian model comparison (BMC) implements an inherent Occam's razor which penalises models in terms of their complexity we assign all models equal prior probabilities $p(\theta)$. Therefore the posterior probability of any model is proportional to model *evidence*:

$$p(D|\theta_i) = \int p(\mathbf{S}|\mathbf{w}, \theta_i)p(\mathbf{w}|\theta_i)d\mathbf{w}, \tag{2}$$

where $\mathbf{S}$ is a set of sequences (data), θ_i a particular chunking model and $\mathbf{w}$ its parameter values. Intuitively, to estimate *evidence* for any model we need to evaluate its complexity as defined by its parameters $\mathbf{w}$ and their probability distributions $p(\mathbf{w}|\theta_i)$, and how well the model fits the data $p(\mathbf{S}|\mathbf{w}, \theta_i)$. By combining the model complexity and data fit we can rank all possible models in terms of their evidence $p(D|\theta_i)$. The model with the greatest evidence is also the model with maximum *a posteriori* probability since we assume equal prior probabilities across models.

Model fit

In Bayesian inference the model fit is defined by the likelihood function which evaluates how likely is that the observed data was generated by a particular model – in our case:

$$p(\mathbf{S}|\theta_i) = p(\mathbf{S}|\mathbf{z}, \mathbf{x}, \theta_i),$$

where $\mathbf{x}$ is a set of n-grams and $\mathbf{z}$ is a set of discrete mappings which define how individual n-grams are combined to encode the observed data $\mathbf{S}$. Intuitively, the likelihood of a model θ_i quantifies how easy or difficult it is to generate all observed sequences using a set of n-grams and mappings as specified by the model.

Commonly, the likelihood of a model is measured in terms of the distance between model predictions and the observed data: for example, we could use a between-sequence distance metric (such as the Levenshtein or Hamming distance) to compute the distances between the observed

sequences $\mathbf{S}$ and the set of sequences defined by a particular model's parameters (n-grams and mappings). However, here we only consider models that are capable of encoding the observed data: e.g. for a set of two sequences $\mathbf{S} = \{ABCD, DBAC\}$ we only consider chunks like $\mathbf{x} = \{AB, CD\}$ or $\mathbf{x} = \{A, B, C, D\}$, but not $\mathbf{x} = \{CA, DD\}$; and the same with mappings. There are two reasons for this: first, the space of possible models that correctly encode the observed sequences is already quite large. For example, in our study we use 14 individual sequences. As any individual 4-item sequence can be encoded with 8 different mappings (see Chunk learning above), it follows that a set of 14 sequences can be encoded with 8^{14} different mappings. Similar combinatorial expansion applies for the number of possible sets of n-grams. Second, the models that cannot even theoretically fit the data are inevitably less likely than models which do. Therefore by constraining ourselves to the subspace of data-matching models we explore the domain of most probable models. This constraint also follows an ecological rationale: chunks are assumed to be inferred from the regularities present in the data, hence there is no reason to consider latent variables that cannot be mapped onto the observed variables.

Model evidence

Model evidence (Eq 2) combines previously described measures: model complexity in terms of the probabilities of its parameters and model fit. Here we only consider models which fit the observed data perfectly: evaluating model evidence is therefore reduced to estimating model complexity for data-fitting models. The model with greatest evidence – and therefore the one with maximum a posterior probability – is the one which encodes the set of observed sequences with the least complex model.

Importantly, the two model parameters – set of n-grams and mappings – make contrasting contributions to model complexity: an optimal model will need to find a trade-off between the number of n-grams it comprises and the complexity of the mappings. For example, a set of four individual uni-grams $\mathbf{x} = \{A, B, C, D\}$ can encode any of the 14 sequences in our task, but all of the mappings need to be maximally complex, each involving four links between the n-grams. Such a model would have a simple set of chunks but would require complex mappings to encode the observed sequences. In the other extreme, consider a model where each individual sequence is encoded with a single four-gram and therefore would require simple mappings (each n-gram to each individual sequence, i.e. four times less complex per sequence than the uni-gram model). However, the such a set of 14 four-grams is by definition more complex and therefore less probable than a set of four simple uni-grams.

The two model parameters – set of n-grams and mappings – can therefore be intuitively thought of as the model's *codes* and the *encoding* it specifies. The Bayesian model comparison mechanism guarantees that the model with the greatest evidence – the optimal model – will

define an ideal trade-off between the complexity of the codes and the encoding it produces. This trade-off can be visualised by displaying the model evidence as a sum of their negative log probabilities: Fig 8B illustrates the trade-off between the codes and the encoding for several possible chunking models given a set of two repeated sequences.
