# Supplementary material for: Sequence learning recodes cortical representations instead of strengthening initial ones
Source: PLoS Comput Biol. 2021 May 24;17(5):e1008969. doi: 10.1371/journal.pcbi.1008969 (PMC8177667; doi:10.1371/journal.pcbi.1008969)
Supplement: S4 Text — (PDF) [file pcbi.1008969.s004.pdf]

S4 Text. Associative learning of overlapping sequences

Worked example

To continue with the example provided in the manuscript: ”The sequences ABCD and BADC cannot be learned simultaneously simply by storing position-item associations, as the resulting set of associations would be equally consistent with the unlearned sequence ABDC.”

When two sequences ABCD and BADC are learned by strengthening item-position associations then (all other variables remaining the same) we end up with equal strengths for the following item-position associations:

$$A - 1,$$
$$A - 2,$$
$$B - 1,$$
$$B - 2,$$
$$C - 3,$$
$$C - 4,$$
$$D - 3,$$
$$D - 4.$$

The resulting weights would also be the result of learning two different sequences ABDC and BACD. In other words, learning the two original sequences would result in eight association weights of equal strength to represent four sequences (ABCD, BADC and ABDC, BACD). Such a learning mechanism would suffer from catastrophic interference with multiple short sequences of overlapping items (like most real-word sequential actions tend to be).
