# Supplementary material for: Sequence learning recodes cortical representations instead of strengthening initial ones
Source: PLoS Comput Biol. 2021 May 24;17(5):e1008969. doi: 10.1371/journal.pcbi.1008969 (PMC8177667; doi:10.1371/journal.pcbi.1008969)
Supplement: S5 Text — (PDF) [file pcbi.1008969.s005.pdf]

S5 Text. Simulation of expected changes in pattern similarity

Our hypothesis about the effects of associative learning assumes noise reduction directly at the level of representational dissimilarity matrix (RDM, Eq 21). Diedrichsen et al. [1] have pointed out that as most distance estimates are based on the product of random variables, the resultant noise variance in the distance estimates gets more complicated than the model we have assumed here. To address this issue we investigated the degree to which the noise should be reduced (or SNR to be increased) in the learned pattern in order for the changes to be detectable in the RDM. Specifically, we carried out a series of simulations to assess how pattern similarity distances change according to the reduction of noise in the activity patterns for the learned sequences. For example, when the measurement noise is already high, a certain amount of noise-reduction in learned sequences would not be visible in the estimated distance measures.

We simulated the predicted effects of associative learning which assume that (1) neural sequence representations remain the same with learning but (2) their SNR changes proportional to the SNR change observed in the behavioural responses. Therefore such change should also be detected in the fMRI data.

Briefly, we first transformed the behavioural change accompanying sequence learning (significant reduction in manual response times, see Behaviour in the Results section of the main manuscript) into the change in the internal representations of sequences. Formally, we assumed that manual response times are proportional to noise in the representation distribution: as noise increases so do the response times. This leads to two representational noise estimates for both unique and repeated sequences which were then transformed into expected voxel responses. The simulated voxel responses where then combined with the estimated fMRI noise using the data from the study's pilot scans. This was carried out using the *CNR/Noise_SD* approach outlined in [2]. The simulated fMRI data was then transformed into voxel RDMs and correlated with stimulus RDMs. Briefly, the steps were as follows:

1. Estimation of population responses according to the sequence representation model given some estimate of the representational noise.
2. Estimation of fMRI noise from unprocessed EPI scans per subject.
3. Combination of simulated population responses with the estimated fMRI noise, resulting in simulated fMRI responses to individual sequences. This step was carried out using the approach and scripts developed by [3], building on previous work by [2].
4. RSA simulation: RSA carried out as described in Methods over simulated fRMI data to estimate a relationship between representational noise and the noise in RDMs.

The full technical details and results of the simulation are presented in the Jupyter Notebook

(*sim_fmri*) at our code repository.

The simulation results are displayed in the plot below outlining the change in the RSA correlation values as a function of representational and measurement (fMRI) noise.

Fig A: Y-axis shows the correlation between stimulus RDM and voxel RDM (Spearman's r) and X-axis shows the amount of noise in the representational model (as represented by the σ noise parameter).

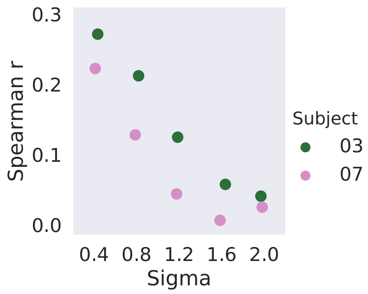

Our simulation shows that we can indeed expect to see a correspondence between representational noise and RSA correlation values as assumed by the SNR hypothesis: RSA correlation values decrease as noise in the sequence representations increases. The individual points om the figure above represent two different fMRI noise estimates corresponding to two subjects we scanned in the piloting phase. The difference between the first two noise parameter values ($\sigma = 0.4$ and $\sigma = 0.8$) corresponds to the estimated noise difference in the novel and learned sequence representations.

References

1. Diedrichsen J, Provost S, Zareamoghaddam H. On the distribution of cross-validated Mahalanobis distances. arXiv. 2016. arXiv:1607.01371.

2. Welvaert M, Rosseel Y. On the definition of signal-to-noise ratio and contrast-to-noise ratio for fMRI data. PLoS ONE. 2013; 11(8). doi:10.1371/journal.pone.0077089.

3. Ellis C, Baldassano C, Schapiro A, Cai M, Cohen, J. Facilitating open-science with realistic fMRI simulation: validation and application. bioRxiv. 2019. doi:10.1101/532424.
