# Supplementary material for: Sequence learning recodes cortical representations instead of strengthening initial ones
Source: PLoS Comput Biol. 2021 May 24;17(5):e1008969. doi: 10.1371/journal.pcbi.1008969 (PMC8177667; doi:10.1371/journal.pcbi.1008969)
Supplement: S6 Text — (PDF) [file pcbi.1008969.s006.pdf]

# S6 Text. Individual sequences used in the task

Four individual items (Gabor patches) are represented with numbers 1 to 4.

$$(3, 1, 4, 2)$$
$$(2, 4, 1, 3)$$
$$(1, 2, 3, 4)$$
$$(4, 2, 1, 3)$$
$$(1, 4, 2, 3)$$
$$(4, 3, 1, 2)$$
$$(4, 1, 3, 2)$$
$$(4, 2, 3, 1)$$
$$(1, 3, 2, 4)$$
$$(1, 2, 4, 3)$$
$$(4, 1, 2, 3)$$
$$(1, 4, 3, 2)$$
$$(1, 3, 4, 2)$$
$$(4, 3, 2, 1)$$
