# Supplementary material for: Sequence learning recodes cortical representations instead of strengthening initial ones
Source: PLoS Comput Biol. 2021 May 24;17(5):e1008969. doi: 10.1371/journal.pcbi.1008969 (PMC8177667; doi:10.1371/journal.pcbi.1008969)

S1 Fig. Anatomical coverage of MRI functional scans

Anatomical coverage of MRI functional scans superimposed on a structural image for a single participant (P13).

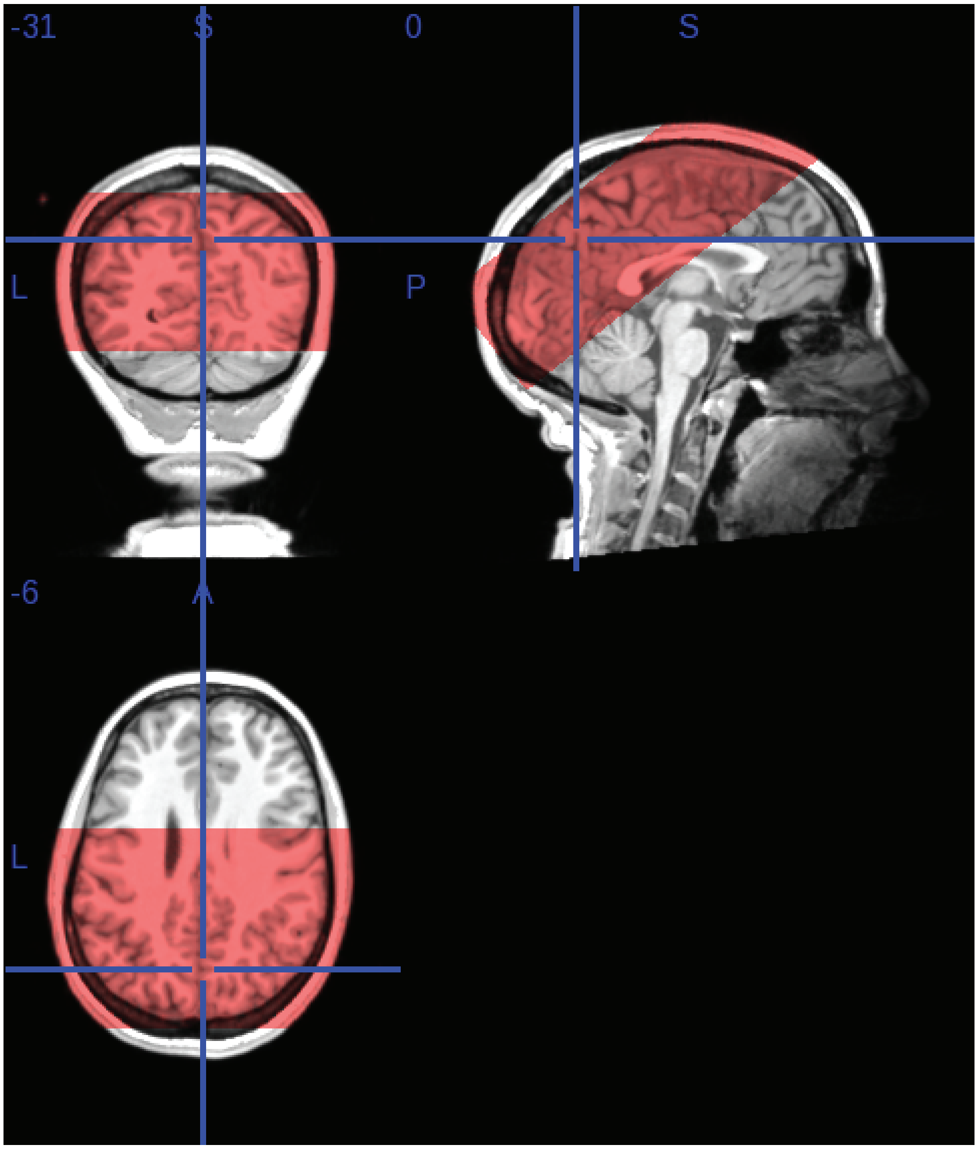

Supplement: S1 Fig — (PDF) [file pcbi.1008969.s007.pdf]
