# Supplementary material for: Sequence learning recodes cortical representations instead of strengthening initial ones
Source: PLoS Comput Biol. 2021 May 24;17(5):e1008969. doi: 10.1371/journal.pcbi.1008969 (PMC8177667; doi:10.1371/journal.pcbi.1008969)
Supplement: Attachment — Submitted filename: responses_to_comments.pdf [file pcbi.1008969.s008.pdf]

Responses to the comments are grouped by individual reviewer. The subsection headings refer to reviewer id/number and the comment number: e.g. '1.6' refers to the 6th comment by Reviewer 1.

1.1

I really like the idea of the paper, the design of the study, the analysis, and the results. It also addresses a current research question in an active research area and does so using state-of-the-art methods, so I would like to see it published in a first class venue such as PLOS Comp Biol.

Thank you for the positive comments and for a thoughtful review of our paper.

However, I found the paper rather difficult to understand. One reason for this might be my lack of familiarity with the relevant fMRI/RSA literature, but I expect that with a better organisation of the manuscript could be made more accessible for a larger audience. In general I think there are some details missing from the introduction and results that should be added to make the exposition clearer.

We have now re-structured both the *Results* and the *Introduction* sections which hopefully will provide extra clarity. In the *Introduction* we have added extra motivation for the design of our experiments and clarified the nature of models test and how the testing is carried out with fMRI data. In the *Results* section we now first compare the models together before proceeding with individual model predictions.

The sheer amount of information required to adequately describe both the models and the fMRI methods made it a challenging manuscript to structure: we hope that this revision has resulted in a more fluent flow of information.

1.2

As someone with some experience in cognitive modelling, I had some problem understanding the role the models played here. To me, a model is usually something that has free parameters that are fitted to the data, but this is not the case here. All models make deterministic predictions a priori. I am not sure if I missed this in the manuscript, but it only became clear in the method section and was necessary in understanding the results. Maybe this could be presented clearer.

The reviewer is right in pointing out that we do not perform model comparison in its traditional sense where parameter values are fit to data. Instead, we contrast two model classes that track different statistics of the environment and hence do not share parameters with each other. However, within each model class — associative and chunking — we chose the parameters so that the individual implementations would correspond to a Bayesian optimal learner. Please see response to comment 1.6 as to whether we could have chosen different particular implementations.

We now highlight this approach in the *Introduction* and have added a separate subsection to the *Discussion* where we discuss whether alternative implementations could lead to substantially different predictions.

1.3

There are also some phrases that seem to be somewhat misleading about the role of the models. For example, line 175 reads: "The resulting RDM of n-gram distances was then fit with neural activity patterns using the RSA method." However, the "fitting" here is the calculation of the distance between two similarity matrices using

rank correlations, which serves here as a measure of model adequacy, and not the more common procedure in which free parameters are adjusted to make the prediction of the model be nearer to the observed data. Similar phrases (i.e., estimated or fitted) can be found in lines 101, 161, and 252.

We agree with the reviewer that 'fit' is not an adequate term here. We have now used the term 'tested' (as in 'model predictions were tested with fMRI data') or 'compared' both here and throughout the manuscript. We only have retained the words 'estimate' and 'fit' in three different contexts where we think it adequately describes the process: (1) estimating the optimal chunking model based on presented sequences, (2) estimating the fMRI noise levels when calculating the noise ceiling, (3) fitting general linear models in the fMRI analysis to estimate the activity levels for individual voxels.

Likewise, Figure 4 refers to "Bayesian model comparison". In most contexts this phrase indicates that different models are compared against the data, but here it is only used to determine the structure of the optimal chunking (i.e., recoding) model before any data is collected.

In this context 'Bayesian model comparison' referred to estimating the optimal chunking model out of many possible ones: the 'data' refers to the set of presented sequences, as in the 'observed variables' based on which the latent model of chunks is inferred. However, we can see how this usage of the term 'data' is confusing in the context of fMRI data. We have now revised the main manuscript so that we now describe 'inferring the optimal chunking structure' and leave the details to the *Methods* section, where we now also explicitly note the distinction between 'data' as presented sequences and fMRI data (*Chunk learning* subsection in *Methods*).

1.4

I generally fund the organisation of the results section somewhat unclear as it jumps between different type of results (i.e., the main RSA results and other fMRI results) and is organised across models. Maybe a clearer organisation in which results are presented one by one for each analysis (and not for each model) might be better. For example, the main result seems to be that only the similarity matrix of the optimal chunking/recoding model shows a significant relationship with the observed similarity matrix, but not for the other three accounts. However, the only graphical representation of this result is Figure 5 which only contrasts 2 models and not all 4.

We have now re-organised the *Results* so that we first present the main result — significant pattern similarity for the recoding model and not for the associative one — and then explore this main finding further model by model. However, the previous model-by-model structure enabled us to introduce the model predictions along the results. After the re-organisation we had to move the model predictions into the separate *Models* section, presented before the *Results*. This has the downside of creating a bit of distance between the predictions of the associative learner (page 4) and the corresponding *Results* section (pages 10-11), but we think the trade-off is worth it.

We have now similarly updated the main results' figure. We did not initially include the item-item and mixture models because: (1) the item-item model was not significant for novel sequences and hence the correlation for the similarity between novel and learned sequences is essentially meaningless (as detailed in the *Associative learning predictions for RSA* subsection in *Methods*); (2) the mixture model is not a learning model, significant correlation would not indicate sequence learning but instead simply mixture of item information (as outlined in the *Methods* section, such null-model is necessary in detecting sequence representations with fMRI, so we used the noise bound for the mixture model as a significance threshold for the actual sequence representation

models).

However, we see the logic that omitting the comparison is confusing, so we have now included the item-item model in the main figure and item-mixture model in the *Supplementary Information* figure. We would prefer not to include the mixture model on the x-axis of the main results figure since this model is only introduced in *Methods* in terms of testing for the null-hypothesis, so including it would require adding to the previous *Models* section before *Results*. Similarly, we would need to change the main figure to indicate that the mixture model is not an a learning model (neither recoding or associative) and it's correlation value doesn't represent evidence for or against learning: this would be confusing without a proper definition of the model itself. Instead, we have now included this a supplementary figure (Fig 11) with the comments above included.

1.5

I also found the additional prediction and analysis with respect to the noise activity the most difficult part of the analysis. Maybe this could be presented after the description of the main RSA results.

As outlined in the response to the previous note, we have now reorganized the *Results* section so that it is presented separately and after the main results, as suggested by the reviewer.

1.6

On the positive side, the implementation of the four sequence representation models appears to be done very thoroughly, and I do not see any large issues. The one thing I think would be interesting to discuss if there is the possibility that additional free parameters (e.g., learning rates or decay) could alter any of the predictions of the models substantially. I do not suggest that such a model should be analysed (it would also be unclear how), but rather whether the deterministic predictions are really the only plausible way associations models could create similarities.

The role of existing and additional model parameters is certainly important.

Associative learning and recoding, as two broad model classes, cover the whole space of possible statistical learning mechanisms of sequence representations. This is because every learning mechanism can be labelled according to the simple distinction whether the dimensionality of representations changes through learning or not [1]. However, within each class of models we could construct different implementations, which would in turn lead to different predictions of similarity between sequences. For example, a chunk recoding model using only 4-item chunks would lead to significantly different predictions.

Since we could not possibly test for all chunk-recoding models we designed our study so that a specific chunk structure would be significantly more likely given the presented sequences, as described in the *Optimal chunking model* subsection in *Methods* (page 34). Therefore, we test for this optimal chunking model, and do not test for alternative ones, since there is no simple way of choosing which specific alternatives should be tested. Models similar to the optimal chunking model would make predictions that are so similar (only differ for a couple of trials worth of data) that distinguishing between them would require statistical power that necessitates several orders of magnitude more data, i.e. the approach would be simply unfeasible given the limits of fMRI. We could have tested for alternative **and** dissimilar chunk models (e.g. a chunking model with only 4-item chunks) but the probability of those models (given the presented sequences) was in our opinion too low — as noted, we designed the experiment so that only one chunking model would dominate the probability mass distribution

(see Fig 7B in the manuscript).

Inverting this process — using fMRI data to infer the chunking structure that is being used by participants — is not easily possible with our task. This is mostly because our study design is suboptimal for such analysis: our sequences were very short, and we only used two individual learned sequences. Longer and more individual learned sequences would drastically increase the statistical power required to tell chunking models apart, see e.g. Acuna et al. [2], Kikumoto and Mayr [3], Thalmann et al. [4]. However, our study was optimized to detect whether the representational change took place or not.

Finally, measurement noise (fMRI noise) in our study could significantly hinder testing our hypotheses. For example, when the measurement noise is already high, a certain amount of noise-reduction in learned sequences would not be visible in the fMRI similarity measures. To address this issue we investigated the degree to which the noise should change in the learned pattern in order for the changes to be detectable in the fMRI data. The results are presented in the *Supplementary Information* (*Simulation of expected changes in pattern similarity*) and show that we can indeed expect to see a correspondence between representational noise and fMRI patterns, given the level of fMRI measurement noise estimated across subjects.

We have now added a paragraph to the *Discussion* section (*Alternative learning models*) which reflects these arguments.

1.7

The description of the results is difficult to understand without reading the method section. Perhaps a few more information about the task could be given. For example, I had the following two questions: What was the task of the participants? Does the task consist of two parts (initial learning phase without fMRI followed by final part with fMRI) or just one part?

We have now added additional task information to the beginning of the new Models section and to the Task figure (Fig 2). We emphasize the task procedure for participants and highlight the whole structure of the experiment: all the behavioural and fMRI measures refer to the participants' performance in the scanner.

Note that it is common, and almost always necessary, to have a practise session outside the scanner in fMRI tasks, since many participants have not been in the scanner before and are unfamiliar with the equipment for responding. In our task participants first had to learn how to use the button box: this involves learning to hold four individual fingers on the buttons without looking (since you cannot see the button box in the scanner). Next, they had to learn which buttons/fingers correspond to which individual items to be able to reproduce the sequences with the button box. We used this practise session to familiarize the participants with two individual sequences which were then labelled as 'learned' at the beginning of the main experiment in the scanner.

1.8

A response time difference of 0.018 seconds (i.e., 18 milliseconds) does not seem to impressive. What is the average RT?

The average time between consecutive key presses for the novel sequences was approximately 0.552 sec, and ca 0.534 sec for the learned sequences. To avoid potential confusion, this number is not the difference in the time it took the participants to start with the response (usual definition of RT), but the difference in the average time between consecutive keypresses, indicating the speed at which recall was performed. We now

report the mean response speeds for both types of stimuli in the corresponding paragraph (*Results*, *Behavioural measures*) of the manuscript. Although the absolute difference in response speeds was not large, the difference was consistent across subjects and hence significant. Figure R1 below shows the mean response speeds for all individual participants; 16 out of 22 responded slower for novel sequences.

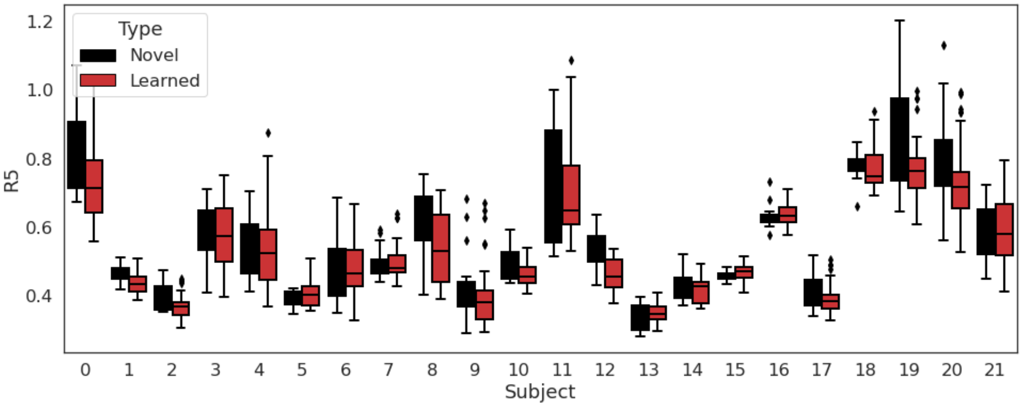

1.9

Fig. 1: I do not know the term "multinomial matrix representation". I understand the figure, but the term is at least uncommon to me.

We agree that the term 'multinomial' isn't necessary for the matrix description and have removed it from Fig 1 legend.

1.10

Fig 2B. Either there is an error in the id column (trial 5 seems to have the wrong id) or I am misunderstanding what it means.

Yes, the Id value was wrong (should have been 3 instead of 2). Thank you for pointing this out.

1.11

Fig. 3 (left): What is the distance measure used to derive the predicted distance between two sequences?

The distance measure for the left matrix on Fig 3 was the Hamming distance, illustrating the associative learning prediction. We have now added this information to the figure legend.

1.12

line 156: "recoded inferred": only one of the two words is necessary.

Thank you — we have now fixed this error.

1.13

Fig. 4: What does "Repeating 2" under "Trial 3" mean?

It refers to the second individual repeating sequence; this is a label as in 'Repeating sequence no. 2'. There is potential confusion here with the ID of the sequence (as displayed on Fig 2, Task) as opposed to which one of the two repeating sequences is displayed, so we have removed the number from the label here (Fig 4).

1.14

lines 373 - 377: It is a bit unclear if the randomisation guarantees that a repeating sequence would always occur in any series of three trials - with the consequence that if one hasn't seen a repeating sequence in the last two trials the next trial would be a repeating sequence with certainty - or whether a repeating sequence would only certainly follow after four non-repeating sequences.

It is the first case — a repeating sequence would always occur in any series of three trials. We have clarified the text accordingly. Please also see our response to comment 3 by Reviewer 2, on whether the structure of the trials could potentially affect learning in our task.

1.15

Line 365: I think it would be good to alert the reader here that the reason why there are only 14 individual sequences is revealed later on. I spent too long wondering why 4! would result in only 14 sequences.

This is a good suggestion, and we have now moved the 'Sequence generation and similarity' subsection in front of the 'Structure of the trials' subsection, so that the reader would be already informed why we only presented 14 individual sequences.

2.0

The manuscript is well written and organized. The models appear well specified and the fMRI data analysis is well conducted, using state of the art tools and methods. Overall, the analyses and their description are of high quality. I also appreciate the authors' commitment to open science by sharing all data and analysis code. The major strength of the present study over most previous ones is the explicit formulation of the learning models, which promises to yield significant additional insight into the neural mechanisms underlying human sequence learning. However, I do have some concerns with the design and interpretation of the results, questioning whether the study truly achieves to provide this additional insight.

Thank you for the positive comments and for a thoughtful review of our paper. We address the issue whether alternative explanations are plausible mostly in comments 2.2 and 2.3, but have now also added a corresponding paragraph to the *Discussion* section (*Alternative learning models*).

2.1

To my knowledge, the neural consequences of visual sequence learning (or visual statistical learning) are commonly reported in primary and ventral visual areas, as also reflected by several papers cited by the authors (e.g. [31-33] in the manuscript; also see de Lange et al., Trends Cogn Sci 2018 for a review). Another line

of evidence suggests the involvement of subcortical structures, such as hippocampus, in statistical learning and positional codes (Hindy et al. 2016; Hsieh et al. 2014). Finally, frontal areas may also encode sequence information in serial order tasks (Berdyyeva and Olson, 2010). Given the prevalence of studies pointing towards sensory, frontal and subcortical areas as key nodes involved in sequence learning, I was surprised by the results in the present manuscript being located exclusively in dorsal visual stream regions (largely parietal cortex; Fig. 5). While the authors state that they want to address the underlying computational mechanism rather than where in the brain effects of learning are found (lines 54-56), I nonetheless believe that the unexpected localization of the results does at least warrants critical evaluation and discussion, also in the context of previous literature.

Indeed, the motivation for the present study is framed in the context of previous work on (visual) sequence/statistical learning and aims to advance our understanding by investigating the underlying computation rather than only a univariate difference in response magnitude – a goal I fully support. However, if the observed neural effects drastically differ in localization, is this goal really achieved in the present study? The authors compare their results to several studies showing modulations in early visual and ventral visual stream areas (e.g. line 195), yet report univariate and RSA results exclusively in dorsal visual areas. Without a convincing explanation for this discrepancy, and why it should not be of concern, it would seem to me that the present results do not provide novel insight into the specific mechanism that was cited to motivate their study.

The reviewer rightly points out that previous literature has observed visual (sequence) learning effects in a range of brain regions not addressed in this study: ventral temporal lobes, basal ganglia, prefrontal cortex. We agree with the reviewer that including such regions to our analysis would have been very relevant. However, as we note in the *Discussion* section of the manuscript, we were unable to do so since our anatomical 'coverage' of the brain provided by the MRI scanner did not reach ventral and subcortical regions. We first explain our focus on the dorsal visual stream, and then relate our results to learning effects observed in different brain regions.

Why did we focus on the dorsal visual areas?

To maximize the statistical power of voxel pattern analysis, we used an MRI resolution of 2 mm (isotropic) with a slice acquisition time of 1.206 sec. Therefore, the height of the MRI acquisition 'box' was quite small, covering only c.a. half of the skull. Figure R2 below shows the alignment of the fMRI 'acquisition box'.

Figure R2

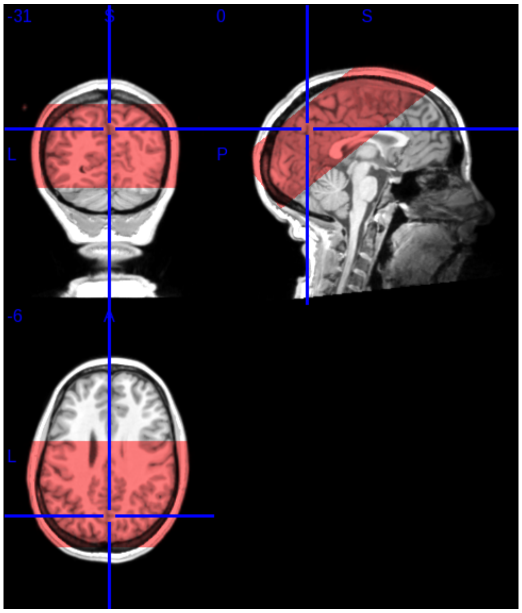

We therefore decided to optimize imaging for the visuo-motor cortical areas, which were most likely to be active in our task, based on past literature.

First, our task is a working memory task: brief presentation of visual stimuli followed by a delay (4.8 sec) and a response (7.2 sec). For each task phase the common anatomical localizations of task representations are all found in the dorsal stream: we would expect the perceptual representations of the stimuli to be localized in the primary visual cortex [5], the delay-phase activity localized in the occipito-parietal and motor-parietal regions [6, 7, 8, 9], and the manual response activity localized in the motor and pre-motor areas [10, 6].

Second, previous studies of sequence learning have observed learning effects in the dorsal visual stream. Statistical learning of sequences has been shown to increase in BOLD SNR [11, 12, 13] and increase fMRI pattern separability [14, 15] in the parietal and occipital regions, similar to visual (perception) learning effects in the primary visual areas [16, 17, 18, 19, 20].

A recent study where mice learned sequential patterns in a short term memory task showed that neurons in the parietal cortex encoded the model of the environment [21] — roughly the anatomical counterpart of the human parietal areas reported in our study. Similarly, previous studies have observed task-dependent sequence representations in the parietal cortex of monkeys [22] and rodents [23].

In sum, our study is by no means the first to report learning effects in the occipito-parietal and pre-motor areas and builds on extensive previous literature. Cortical localisation of the effects depends heavily on the task and stimulus modality: since our task required manual recall of visual stimuli we should expect neural populations in dorsal regions to be heavily involved both in task representation and learning. We now highlight these studies in more detail in both the *Introduction* and *Discussion* sections.

Based on our task and previous literature, we therefore chose to orient our acquisition box so that it would focus on the visual, parietal, motor and pre-motor (prefrontal) regions (Figure R2). This meant that our MRI coverage did not reach the ventral or deep subcortical regions, such as the hippocampal formation or the mediotemporal lobe. In order to get a good SNR for the subcortical signal one needs to change the angle of MRI slice acquisition [24, 25], which would effectively rule out most of the cortical areas discussed above. Given the complexity of the analysis in our task we opted for a safer route of optimizing for the acquisition of cortical signals.

How do our results relate to other brain areas in the context of previous learning studies?

Although we observe learning effects in the dorsal visual stream we think it's very likely that multiple brain regions are involved in sequence learning. Our results do not exclude localizing learning effects to other brain areas: in fact we think it is very likely that similar effects could be observed in other brain regions, given the fundamental role of hippocampus and striatum in structure learning.

For example, a well-established model of neural learning [26, 27] proposes that the initial hippocampal-dependent associative memories are recoded as they undergo consolidation resulting in more efficient cortical representations [28, 29]. Specifically, while the initial encoding of individual events or items into novel sequences is facilitated by the hippocampal formation [30, 31, 32], learning modulates the parallel cortical representations so that these representations can become independent of the hippocampus over time: humans and animals with hippocampal lesions are unable to learn new sequences but are able to recall already learned ones [31, 33]. This suggests a dissociation between the hippocampal and cortical representations: both are created simultaneously but affected differently by learning. However, it is unclear how this recoding happens for novel sequences, what is the recoding computation, and exactly how do the neural codes for novel and learned sequences differ. The

objective of this study was to address these questions by focussing at the change of representation at the cortical level. Again, we now discuss the results of our study in the wider context of neural learning systems in the *Discussion* section.

In sum, we feel there is no discrepancy between our results and studies which have observed learning effects in different regions. The anatomical localization of our results is expected given we used a working memory task with manual recall: the same brain regions have been reported to encode learning effects in a wide range of learning and sequence processing tasks as highlighted above. Second, our results do not exclude parallel learning mechanisms: there is overwhelming evidence that learning proceeds simultaneously using multiple neural pathways. The effects of learning in cortical areas have been shown to be mediated by the hippocampus and the basal ganglia: we think that similar mechanisms are very likely in our task. However, the limitations of fMRI measurement meant that we had to focus on some part of the cortex, but we believe that our method would likely yield similar results for the HC/BG areas, which is the focus of our ongoing research.

We now address these issues in a separate section in *Discussion* ('Multiple and parallel systems for sequence learning'), which will hopefully provide necessary context for interpreting our results in the light of a larger body of learning literature.

2.2

Relatedly, the specifics of the experiment design and the cortical areas reported by the authors may in fact suggest an alternative explanation for the results. In the task performed by the participants, the recall of stimulus order, responding to novel compared to learned sequence is more difficult. Accordingly, also behavioral results show slower responses to the more difficult to recall novel sequences. Arguably, trials with novel sequences require more attention, likely over a longer duration, to be devoted to the stimuli. The univariate fMRI results are in line with this interpretation, by showing enhanced neural responses to novel compared to learned sequences; i.e., possibly an enhanced neural response to novel sequences due to attention. Moreover, there appears to be an appreciable overlap of the results reported in Fig. 5, with posterior parts of the default mode and attention related networks. Combined this evidence suggests an interpretation stressing the role of attention allocation and task difficulty as underlying (at least partially) the observed effects. Therefore, to me, it appears unclear what neural mechanism is reflected in the present results. This is a notable limitation, which should be critically evaluated by the authors, or if possible be addressed with additional analyses – although, unfortunately I do currently not see how the existing data could directly answer this concern.

The reviewer proposes that the learning effects could be explained by differential allocation of attention between novel and learned stimuli. This is a common question raised with learning tasks: we first formalize this hypothesis in terms of our task and then consider whether it provides an alternative explanation.

A classic definition of attention in the context of learning is outlined by Peter Dayan and colleagues in their seminal paper 'Learning and selective attention' [34]. According to this model, attention refers to differences in neural resource allocation induced by learning. In other words, an optimal learner should allocate resources to stimuli proportional to the amount of learning they provide, where learning is quantified as a change in participants' internal model. This normative account defines attention as differential resource allocation which is computationally sensible by itself and not a response to any fixed limit or constraint.

To make this model of attention explicit in the context of our task, consider how should the participants allocate encoding resources when presented with a 'learned' or a 'novel' sequence. A learned sequence had been presented

12 times at the beginning of our experiment and therefore would be roughly 12 times more probable at that point. The resource allocated can be formalized in terms of bits required to encode the stimuli S, which for the optimal learner is inversely proportional to their probability: $H(S) = -\int p(S)\log(p(S))$.

Therefore, we would expect to see differential resource allocation (attention) in encoding novel and learned sequences, since learned sequences have higher prior probability and thus provide relatively little new information about the environment compared to novel ones. Contrastingly, novel sequences require more bits to encode since they provide more information; intuitively, their probability mass is more 'spread out'.

Since the probability of learned sequences is necessarily higher for any learning mechanism (including associative and chunk learner) it follows that any learning account would predict such different resource requirements.

However, as outlined in the manuscript, our hypotheses were designed to go beyond the simple distinction between novel and learned stimuli – instead, we test whether a change in representations occurs. In other words, both our models predict similar differences in of resource allocation but make dissociable predictions in terms of the dimensionality of neural responses. The associative learner would not change the representations (same dimensionality) while recoding would: this distinction is orthogonal to that of resource requirements.

Therefore, the effects of 'attention' in terms of differential resource allocation — differences in univariate BOLD response, processing times and participants' estimates of task difficulty — should be expected for any learning mechanism, independent of its underlying computations. The lack of such markers should be alarming for any learning experiment. However, attentional predictions would only tell us of how much resource should we allocate for novel vs learned stimuli, but it does not tell us whether we should change the code or not.

A parallel stream of studies have explored the effects of attention with paradigms where it is artificially enforced: e.g. using distractors or forcing participants to switch between multiply present stimuli. In such experiments there are usually 'attended' stimuli, as defined by instructions. For example, Richter and de Lange [16] studied the effects of 'attention' in statistical learning of visual features, using a task where participants were instructed to ignore either one or the other of concurrently present stimulus features. The authors found no attenuation of the BOLD response for the unattended features in primary visual areas. However, in this task the model of the environment changes with the attend/ignore switch and there is no reason why regularities in one domain should predict resource allocation in another. Contrastingly, in our task any attend/ignore switch can only result from the statistics of the stimuli themselves and differences in resource allocation are thus entirely predictable by learning.

In sum, attentional effects as described by the reviewer should be present in any learning task. However, the results of our study — change in the representation of sequences by learning — are orthogonal to these effects and not in any way dependent or explained by differences in resource allocation. As we argue in the *Introduction* section, such univariate effects should be expected for any learning mechanism, and the simple of detection of such markers — e.g. decrease in the BOLD signal or reaction times — does not allow dissociating between individual learning mechanisms. Instead, we test for the change of dimensionality in sequence representations.

Note, that 'attention' takes a slightly different meaning when discussing the possibility that participants might stop encoding the stimuli once they had been identified as the familiar 'learned' sequences and "some other simpler process might take over" — we discuss such possibilities in detail in the response for the next comment (2.3).

2.3

The critical question is then whether differences in attention or related effects, brought about by differences in task difficulty and task demands between the conditions of interest, could also account for (apparent) changes in neural representations; the RSA results at the heart of the manuscript. To me this seems possible, albeit less straight forward than for the univariate results. Nonetheless, one can speculate.

If I understand correctly, the authors constrained sequence selection (maximized differences between sequences) and pseudo-randomized the order of trials (2 learned, 1 novel sequence for every set of 3 trials). This maximization of differences between sequences and 2nd order regularity across trials probably makes it possible for participants to rapidly and with high probably detect learned sequences after the first stimulus of a sequence is presented. I believe that the third sequence in a set of three trials can even be perfectly predicted before stimulus onset, if 1 learned and 1 novel trial have been seen so far. Even without this 2nd order regularity, learned sequences can be detected rapidly. That is, if the first stimulus belongs to one of the two learned sequence chances are very high that the sequence is the learned sequence; because only 1/3 of sequences are novel sequences and of those only 1/4 start with any one particular stimulus, while on the other hand 2/3 of trials are learned sequences of which each starts with a different stimulus. Thus, for learned sequences participants can rapidly disengage attention after recognizing the specific learned sequence as e.g. "sequence 1", possibly already upon seeing the first stimulus in a sequence. Then participants can simply recall the required responses before preparing to perform the associated action. Note that no attention to subsequent stimuli is necessary in most cases. On the other hand, novel sequences require sustained attention to the stimulus sequence itself, do not allow observers to preemptively disengage, demand storing the associated stimuli in working memory, prepare a novel motor plan, etc. In other words, many aspects differ in how observers may respond to learned compared to novel sequences. Importantly, many of these differences do not appear to be related to how learning changes the representation of a sequence itself or the representation of stimuli in a sequence, but rather are secondary consequences of having learned sequences in this task, and of making use of what has been learned.

It seems possible that one of these differences, or a combination thereof, may result in a better fit of the recoding model compared to the associative learning model. Consider that a learned sequence can be recognized rapidly (e.g. as "sequence 1") and attention disengaged from the stimuli, only requiring the participant to recall the motor sequence to be performed. The associated representation of this recognition and the secondary processes outlined above would be highly unlikely to resemble the predictions of the associative learning model, but rather resemble a new "recoded" representation. However, this does not seem to me to indicate that the representation of the sequence has necessarily been recoded, but another secondary process may mask as a recoded sequence representation.

Even so, it is interesting how these secondary consequences modulate neural responses – i.e., I do not want to argue that the results are uninteresting. Rather, the problem is again that we do not seem to know which mechanism, modulation, strategy, etc. is underlying the present results. In a study that aims to narrow down the precise neural mechanism underlying sequence learning this uncertainty about which process may underpin the observed results is, in my opinion, a crucial limitation worth discussing.

The reviewer here raises two important points, related to the previous comment about the role of attention in our task:

(1) The participants could anticipate a learned sequence and 'disengage', and similarly assign more attention/resources to the novel sequences. Therefore, "these differences do not appear to be related to how learning changes the representation of a sequence itself".

As described in our response to the previous question, according to a normative model of attention [34] such differential resource allocation should be expected for any learning mechanism and would result in univariate effects of encoding cost, perceived task difficulty, response time, etc. The reviewer is right in pointing out that such effects can not indicate whether a representational change has taken place.

Instead, we use a representational similarity prediction which is invariant to changes in mean activity. The RSA does not depend on or take account the differences in average activity between novel and learned stimuli (in the same way as correlation is independent of the absolute values of the signal). Therefore, our predictions are independent of any univariate differences between the learned/novel stimuli and directly test for a particular representation.

However, this raises a secondary question: if participants do not need to attend or engage with the learned sequences (once they have been identified as such) could "another secondary process may mask as a recoded sequence representation." For example, after seeing the first two items the rest of the sequence is highly predictable and therefore some kind of pattern-completion or chaining mechanism could retrieve the whole sequence automatically. Next, we discuss whether such alternative mechanisms are plausible and can they be dissociated from our hypotheses.

2) Can a simpler process than recoding account for the similarity between learned and novel sequences?

Let's first formalize the identify-and-disengage hypothesis. Any 'disengagement' from attending the presented stimulus can only happen after the complete sequence has been retrieved, otherwise accurate recall would not be possible later. Thus, such an identification must implement a pattern completion mechanism: infer the whole sequence from partial information. Recurrent neural networks have been commonly used as a model of such a mechanism: for example, the pattern-completion computations performed in CA3 area of the hippocampus are thought to be supported by the overwhelmingly recurrent local connections [35].

However, such a pattern completion model requires a mapping function that takes the partial input and returns a full representation. This is usually formalized in the associative learning literature in terms of transitional probabilities between successive events or items [36] and the retrieval of a whole pattern as a Markov chain of arbitrary order (e.g. ABCD as $p(A)p(B|A)p(C|B)p(D|C)$ as a 1st order Markov Chain). The transitional probabilities themselves are inferred from co-occurrence of individual items. If we constrain the transitional probabilities only to adjacent items (1st order chain) then the mapping function is equivalent to inferring two-item chunks, where the chunk probability for AB is equal to the transitional probability of B following A. In the case of discrete variables, pattern completion is formally and representationally equivalent to inferring chunks: in both cases there needs to be a latent variable which encodes the co-occurrence probabilities for individual items.

In our view the identification-and-disengage mechanism is only possible using a latent variable that maps onto a longer sequence. This model can be called pattern completion or chunking — in our manuscript we use the neutral term recoding.

Similarly, faster identification can be also supported by associative learning: frequently occurring associations (learned sequences) require fewer bits to encode compared to novel ones and therefore can be encoded and retrieved will less resources. This, in turn, leads to participants 'disengaging' from learned sequences faster compared to novel ones.

In sum, we believe that any quick identification of the learned sequences — either due to the predictability of the trials or from partial information — requires a process which is identical to learning. In case of sequences,

it is either formally equivalent to mapping observed variables to latent ones (recoding) or simply reflects the modified association strengths. The whole purpose of learning is to make the representation of predictable events more efficient. Such compression or 'sharpening' would be impossible without a specific learning function: from an information-theoretic viewpoint this would be equal to a 'free lunch' where information is somehow stored without a cost.

Instead, the main question of our study is of representational change: whether the efficient codes for predictable sequences are newly inferred chunks (recoding) or simply modified association strengths (no new codes necessary). We show that the results of the fMRI pattern similarity analysis support the recoding model and that recoding also avoids significant interference between learned sequences.

2.4

Related to the points above, one may also wonder whether the discrepancy in results compared to previous studies could also be a consequence of differences in stimulus duration. Compare for example the fast-paced statistical learning in [31-33] (stimulus presentation <=500ms), which resulted in reduced sensory responses to learned sequences in early and ventral visual areas, with the slow paced presentation (2.4s) in the present study. Pacing could thus be an important characteristic relevant for determining which coding strategy is employed. If this is the case, it would limit the generalization of the conclusions we can draw from the present study.

We agree with the reviewer that the fast-paced statistical learning mechanisms observed in [31-33] are likely distinct from the learning effects observed in our task. Not only did we use a much slower presentation rate, our task is also a working memory task, with a significant delay (4.8 s) after the presentation.

As discussed in previous comments and in our *Discussion* section: adaption/learning happens at all timescales in parallel. Statistical regularities need to be tracked both at the level of milliseconds, and days and weeks [37, 38, 39]. Previous studies have suggested that the temporal window of learning in our task is measured in several seconds: when the repeating sequences are spaced minutes apart, little or no learning happens [40]. Therefore, in terms of cognitive psychology, we think it's likely that the change of representations in our study happens at the level of working memory: the initial working memory representations are associative and then recoded with chunks after learning.

We agree with the reviewer that it's important to distinguish between learning at different time scales and also at the level of statistical variables. Our task mimics a naturalistic working memory setting where multiple overlapping sequences are processed in succession (reading, speaking, driving, following a recipe) and therefore both the anatomical localization and the tracked statistics differ markedly from perceptual learning tasks [31-33]. In a different task there could be little motivation to switch from associative codes to latent variables, because recoding provides little advantage. However, in our task there is a significant switching advantage since associative strengthening results in serious interference. (Note that when we say 'switching' we don't mean that one of the learning mechanisms stops and the other one starts: rather it's an internal choice of what level of representation is useful for a given task.) Contrastingly, recoding does not seem to provide any theoretical advantage for tracking the statistics of oriented lines: there seems to be no categorical representation of orientation in humans [41], instead, latent variables are inferred at the level of visual segments or objects.

In sum, our results are specific to sequences and learning from working memory. We now highlight the distinctions discussed above more explicitly in the *Discussion* section of our manuscript.

2.5

In lines 118-124, the authors explain the rationale of the test of the associative learning model. I understand the incentive to use RSA here as well. However, it seems to me that a decoding approach (e.g. SVM), comparing decoding accuracy of novel compared to learned sequences, may have provided a more powerful approach, assuming appropriate training data would have been acquired. It might be worth explaining why RSA would be preferable over a decoding analysis (or at least equally suited) to assess predictions of the associative learning model. Otherwise, it may appear to the reader that the study design and analysis procedure is optimized for the recoding model.

The main reason of adopting the RSA approach is that it allows distinguishing between different models of sequence representation, which would be significantly more complicated (if not impossible) with linear classification.

Specifically, to test our hypotheses of representational change through learning, we need to establish how both novel and learned sequences are represented in terms of neural activity patterns. For this purpose we defined several associative models (item-position, item-item, mixture) and a chunking model. As a worked example, lets assume we want to find evidence for associative item-position codes. This depends on calculating predicted distances between sequences: for three sequences used in our study (3124, 2413, 4312) the normalized item-position distances are as follows:

d(3124, 2413) = 1,

d(3124, 4312) = 1,

d(2413, 4312) = 1/4,

d(3124, 3124) = 0,

where the distance values indicate the proportion of associations which are not shared (e.g. 1 = none shared, 0 = all shared). It is clear that the model predictions cannot be split into discrete equidistant classes, such as in standard linear classification (e.g. binary feature labels). Instead, if linear classification is to be used, it needs to minimally implement multi-class voting (14 individual classes) with weights attached to individual labels. RSA provides a significantly less complex and transparent method of achieving our goal. Furthermore, RSA provides a straightforward way of estimating how much variance in brain data – given the noise level – is expected to be explained by an ideal 'true' model (noise ceiling). This is extremely useful given the prevalence of measurement noise in fMRI data (up to 80-95% of the variance depending on the task [42]).

Finally, as discussed at length in previous comments, a binary novel vs. learned sequences decoding would measure the simple differential resource prediction of any learning model, and not be sensitive to changes in representation.

2.6

The authors conclude "humans follow an optimal sequence learning strategy and recode initial sequence representations into more efficient chunks" (line 314-315). What is meant here by "optimal"? I am not sure that I quite see how the data support that an optimal strategy is necessarily used, but only that recoding fits the current data better than the associative learning model and the null model. I do understand that practical limitations constrain which models can be implemented, but (at least in my understanding) it has not been tested whether

an optimal learning strategy is indeed used.

Here we meant "optimal" in the sense of given the choice between associative strengthening and recoding, an optimal learner should choose recoding since it provides significantly more efficient storage of sequences. As we show in the manuscript, learning by strengthening of associations would quickly lead to severe interference and has a hard limit on the number of to-be-learned sequences (5, in the case of 4-item overlapping sequences, like in our task). Recoding removes such interference and provides potentially infinite memory: e.g. people's use of language or music suggests there is no hard limit on the long term memory of overlapping sequences.

However, as the reviewer points out, we don't quantify this optimality in any way, and it assumes a choice between two specific learning mechanisms. Hence, we have now replaced "optimal" with "efficient" both in that particular sentence and in similar contexts.

2.7

Lines 123-124 first introduce the dorsal visual stream as a ROI. This choice of ROI should be supported or introduced in some fashion.

We have now included the motivation for the focus on the dorsal stream in the *Discussion* section as highlighted in a previous comment (2.1). We also added a cross-reference to the dorsal focus paragraph to the *Results* section, where the ROI selection is first mentioned (*fMRI evidence for learning models*, first paragraph).

2.8

Lines 126-127 state that "We found no evidence for the first associative learning prediction: novel and repeating sequences were not encoded similarly in any of the brain regions". Would it be possible to clarify which results the authors are referring to here?

This sentence referred to the lack of similarity between novel and learned sequences as predicted by the associative model. It also references that the similarity prediction is only one of the hypotheses of associative learning: it also predicts that learned sequences should have less noise. But since the latter test is not possible (since no common representation between novel and learned) we have now rephrased this, so that is simply reads *"We found no evidence for the associative learning prediction that novel and repeating sequences are encoded with same representations in any of the brain regions."*

2.9

In addition to the lower and upper noise ceiling for the model results, it might be helpful to mention the variance explained by the models.

The variance explained by the models is indicated on the y-axis of the plot. For example, Fig 5, first subplot on the left (panel A) shows the model evidence and the noise ceiling as measured with Spearman's rank correlation, which in turn can be squared to indicate to the proportion of variance explained in terms of ranks (as the Spearman's coefficient is simply the Pearson's correlation coefficient over the ranks of the data). However, since we specifically used the Spearman's rank correlation as the measure of similarity between model predictions and fMRI pattern data, we think the results should be reported without transforming the correlation coefficient into R^2.

2.10

Important additional MR sequence parameters, such as the multi-band factor, etc., would be a good addition to the Methods section (around line 782). This would better allow the reader to evaluate potential shortcomings of the utilized sequence.

We now report the additional parameters in the *fMRI data acquisition and pre-processing* sub-section of *Methods*: we used a multi-band factor of 2, partial Fourier 7/8, and applied no parallel imaging techniques (i.e. partial Fourier only in the form of in-plane acceleration).

2.11

Has the MR sequence been evaluated for slice leakage?

No explicit evaluation of slice leakage was performed, however, with a multi-band factor of only 2 and no GRAPPA in-plane acceleration the sequence is not expected to be vulnerable to significant false positive activation due to slice leakage, see:

Todd, N., Moeller, S., Auerbach, E. J., Yacoub, E., Flandin, G., & Weiskopf, N. (2016). Evaluation of 2D multiband EPI imaging for high-resolution, whole-brain, task-based fMRI studies at 3T: Sensitivity and slice leakage artifacts. NeuroImage, 124, 32–42. `https://doi.org/10.1016/j.neuroimage.2015.08.056`

The key conclusion of the paper above is that "(2) false-positive activation arising when BOLD signal changes due to true positive activation in one slice leak into other simultaneously excited slices can occur when using multiband factors of 4 or higher combined with in-plane accelerations" (Conclusions, page 41). The sequence we used is therefore not expected to suffer from this.

2.12

Was mean scaling performed as part of the univariate fMRI analysis pipeline?

No, mean scaling was not performed for the univariate analysis, as opposed to pattern similarity/distance analyses. This was not explicitly mentioned in the *Methods* section, and we have now corrected this oversight by clarifying this in the *Model-free fMRI analyses* subsection in *Methods*.

2.13

Several figures (e.g. Fig. 5, Fig 9) would benefit from labels on the y-axis and the colorbar.

We have now added the labels and the color-bars to the figures themselves, instead of relying on the figure legends.

2.14

The authors mention that in $\frac{1}{4}$ of trials the recall phase was omitted. Was this data used in any specific manner? What was the intention behind this design choice? The stated rationale, "to ensure a sufficient degree of decorrelation between the estimates of the BOLD signal for the delay and recall phases" would have been possible to achieve by using a variable duration for the delay window.

The intention behind this choice was solely to decorrelate the BOLD estimates of individual task phases, as mentioned in the manuscript. We could have used a variable duration for the delay phase, as the reviewer suggests, but this would have resulted in unequal recall conditions: the length of the delay is potentially going to affect recall performance, as longer delay would theoretically make recall harder. Therefore, we opted to keep the phase lengths fixed across all trials.

2.15

I could not find the fMRI dataset under the link provided in the manuscript. I assume that the authors will publish the dataset upon publication?

Currently, publishing the dataset requires also peer-reviewed documentation of the data and the results (i.e. a linked publication). Therefore, once we have received conditional acceptance for our manuscript we can link the data immediately. This can be verified by the editor and the reviewers before making the manuscript public on the PLOS website.

2.16

I can confirm that I could access the code shared on gitlab. The code seems well written and documented. Sharing code in a well readable format is much appreciated. That said, I did not have time to adequately review the code or test it, also due to the problems with data access.

2.17

Reviewer #2: No: I could not access the MRI dataset using the link in the manuscript, and I did not notice any separate numerical data underlying the figures. However, this could also have been an oversight/problem on my part.

The numerical data underlying the figures were extracted from the MRI dataset as follows. First, statistical estimates were extracted for every participant and stored as comma separated text files. The participant-level data was then aggregated into a single data file (CSV format), which is used to perform the group level calculations and create figures. However, we appreciate that the full workflow is not replicable until the BIDS dataset is available (see response to comment 2.15).

3.1

An additional comment that I have is that it could be useful to include a brief discussion about the possible role of long-term memory in these results. One question that I kept asking myself is whether part of the results may be explained as a consequence of a shift from working memory to long-term memory in trials with repeated sequences, while the novel sequences would always have to rely solely on working memory. I don't know whether long-term memory processes should be seen as an alternative explanation or rather as a possible mechanism behind the recoding hypothesis. Some discussion on this would be helpful.

Sequence learning in working memory tasks such as ours has been described as a paradigmatic example of interaction between working memory and long-term memory [43]. This model assumes that the function of working memory (WM) is to encode lower-level input in terms of the latent variables represented in long-term

memory (LTM). For example, auditory-verbal WM encodes auditory input using phonemes and syllables stored in LTM. The inferred phonemes and syllables are in turn used to infer words and phrases, and so forth. Such conditional inference of structured representations is common for any learning mechanism — 'working memory' here simply denotes inference in a time-window of several seconds, instead of milliseconds or days.

We think that the involvement of LTM in our task is inevitable: the advantage for repeating/learned sequences in our task has been shown to last for several weeks [40]. However, it is an open question which biological systems and at which level support such learning: we now discuss potential candidates at systems level (hippocampal vs cortical learning) in our extended *Discussion* section.

References

[1] Peiran Gao, Eric Trautmann, Byron M. Yu, Gopal Santhanam, Stephen I. Ryu, Krishna V. Shenoy, and Surya Ganguli. A theory of multineuronal dimensionality, dynamics and measurement. *bioRxiv*, page 214262, 2017. doi: 10.1101/214262.

[2] Daniel E. Acuna, Nicholas F. Wymbs, Chelsea A. Reynolds, Nathalie Picard, Robert S. Turner, Peter L. Strick, Scott T. Grafton, and Konrad P. Kording. Multifaceted aspects of chunking enable robust algorithms. *Journal of Neurophysiology*, 112(8):1849–1856, 2014. doi: 10.1152/jn.00028.2014.

[3] Atsushi Kikumoto and Ulrich Mayr. Decoding hierarchical control of sequential behavior in oscillatory EEG activity. *eLife*, 7:1–36, 2018. doi: 10.7554/elife.38550.

[4] Mirko Thalmann, Alessandra S. Souza, and Klaus Oberauer. How does chunking help working memory? *Journal of Experimental Psychology: Learning, Memory, and Cognition*, 38(5):1206–1220, 2018. doi: 10.1037/xlm0000578.

[5] R Maloney. The basis of orientation decoding in human primary visual cortex: fine- or coarse-scale biases? *Journal of neurophysiology*, pages 1–13, 2014. doi: 10.1152/jn.00196.2014.

[6] Freek van Ede, Sammi R. Chekroud, Mark G. Stokes, and Anna C. Nobre. Concurrent visual and motor selection during visual working memory guided action. *Nature Neuroscience*, 22(3):477–483, 2019. doi: 10.1038/s41593-018-0335-6.

[7] Carmen Wong and Stephen G. Lomber. Stable Delay Period Representations in the Posterior Parietal Cortex Facilitate Working-Memory-Guided Obstacle Negotiation. *Current Biology*, 29(1):70–80.e3, 2019. doi: 10.1016/j.cub.2018.11.021.

[8] Simon Nikolas Jacob, Daniel Hähnke, and Andreas Nieder. Structuring of Abstract Working Memory Content by Fronto-parietal Synchrony in Primate Cortex. *Neuron*, 99(3):588–597.e5, 2018. doi: 10.1016/j.neuron.2018.07.025.

[9] Katherine C Bettencourt and Yaoda Xu. Decoding the content of visual short-term memory under distraction in occipital and parietal areas. *Nature Neuroscience*, 19(1):150–157, 2015. doi: 10.1038/nn.4174.

[10] Atsushi Yokoi and Jörn Diedrichsen. Neural Organization of Hierarchical Motor Sequence Representations in the Human Neocortex. *Neuron*, pages 1–13, 2019. doi: 10.1016/j.neuron.2019.06.017.

[11] Rui Wang, Yuan Shen, Peter Tino, Andrew Welchman, and Zoe Kourtzi. Learning predictive statistics: strategies and brain mechanisms. *The Journal of Neuroscience*, 37(35):0144–17, 2017. ISSN 0270-6474. doi: 10.1523/JNEUROSCI.0144-17.2017. URL `http://www.jneurosci.org/lookup/doi/10.1523/JNEUROSCI.0144-17.2017`.

[12] Joseph Giorgio, Vasilis M. Karlaftis, Rui Wang, Yuan Shen, Peter Tino, Andrew Welchman, and Zoe Kourtzi. Functional brain networks for learning predictive statistics. *Cortex*, 107:204–219, 2018. ISSN 19738102. doi: 10.1016/j.cortex.2017.08.014.

[13] Jeffrey P Gavornik and Mark F Bear. Learned spatiotemporal sequence recognition and prediction in primary visual cortex. *Nature Neuroscience*, 17(5):732–737, 2014. ISSN 1097-6256. doi: 10.1038/nn.3683. URL `http://www.nature.com/doifinder/10.1038/nn.3683`.

[14] Basile Pinsard, Arnaud Boutin, Ella Gabitov, Ovidiu Lungu, Habib Benali, and Julien Doyon. Consolidation alters motor sequence-specific distributed representations. *eLife*, 8:1–29, 2019. doi: 10.7554/elife.39324.

[15] Tobias Wiestler and Jörn Diedrichsen. Skill learning strengthens cortical representations of motor sequences. *eLife*, 2:1–20, 2013. doi: 10.7554/elife.00801.

[16] David Richter and Floris P. de Lange. Statistical learning attenuates visual activity only for attended stimuli. *eLife*, 8:1–27, 2019. doi: 10.7554/elife.47869.

[17] Caroline D. B. Luft, Alan Meeson, Andrew E. Welchman, and Zoe Kourtzi. Decoding the future from past experience: learning shapes predictions in early visual cortex. *Journal of Neurophysiology*, 113(9): 3159–3171, 2015. doi: 10.1152/jn.00753.2014.

[18] Shu-Guang Kuai, Dennis Levi, and Zoe Kourtzi. Learning Optimizes Decision Templates in the Human Visual Cortex. *Current Biology*, 23(18):1799–1804, 2013. doi: 10.1016/j.cub.2013.07.052.

[19] R. Guidotti, C. Del Gratta, A. Baldassarre, G. L. Romani, and M. Corbetta. Visual Learning Induces Changes in Resting-State fMRI Multivariate Pattern of Information. *Journal of Neuroscience*, 35(27): 9786–9798, 2015. doi: 10.1523/jneurosci.3920-14.2015.

[20] Janneke F M Jehee, Sam Ling, Jascha D Swisher, R. S. van Bergen, and Frank Tong. Perceptual Learning Selectively Refines Orientation Representations in Early Visual Cortex. *Journal of Neuroscience*, 32(47): 16747–16753, 2012. doi: 10.1523/jneurosci.6112-11.2012.

[21] Athena Akrami, Charles D. Kopec, Mathew E. Diamond, and Carlos D. Brody. Posterior parietal cortex represents sensory history and mediates its effects on behaviour. *Nature*, 554(7692):368–372, 2018. doi: 10.1038/nature25510.

[22] David A. Crowe, Bruno B. Averbeck, and Matthew V. Chafee. Rapid sequences of population activity patterns dynamically encode task-critical spatial information in parietal cortex. *Journal of Neuroscience*, 30(35):11640–11653, 2010. doi: 10.1523/jneurosci.0954-10.2010.

[23] Christopher D. Harvey, Philip Coen, and David W. Tank. Choice-specific sequences in parietal cortex during a virtual-navigation decision task. *Nature*, 484(7392):62–68, 2012. doi: 10.1038/nature10918.

[24] Matthias Nau. Functional imaging of the human mediotemporal lobe: A neuroscientist's guide to fMRI pulse sequence optimization. 2019.

[25] C Olman, L Davachi, and S Inati. Distortion and Signal Loss in Medial Temporal Lobe. *PLoS ONE*, 4 (12):e8160, 2009. doi: 10.1371/journal.pone.0008160.

[26] James L McClelland, Bruce L McNaughton, and Randall C O'Reilly. Why there are complementary learning systems in the hippocampus and neocortex: Insights from the successes and failures of connectionist models of learning and memory. *Psychological Review*, 102(3):419–457, 1995. doi: 10.1037/0033-295x.102.3.419.

[27] A Treves and E Rolls. Computational analysis of the role of the hippocampus in memory. *Hippocampus*, 4(3):374–91, 1994. doi: 10.1002/hipo.450040319.

[28] Sam McKenzie, Christopher S. Keene, Anja Farovik, John Bladon, Ryan Place, Robert Komorowski, and Howard Eichenbaum. Representation of memories in the cortical–hippocampal system: Results from the application of population similarity analyses. *Neurobiology of Learning and Memory*, 134:178–191, 2016. doi: 10.1016/j.nlm.2015.12.008.

[29] Susumu Tonegawa, Mark D. Morrissey, and Takashi Kitamura. The role of engram cells in the systems consolidation of memory. *Nature Reviews Neuroscience*, 19(8):485–498, 2018. doi: 10.1038/s41583-018-0031-2.

[30] Arthur P. Shimamura, Jeri S. Janowsky, and Larry R. Squire. Memory for the temporal order of events in patients with frontal lobe lesions and amnesic patients. *Neuropsychologia*, 28(8):803–813, 1990. doi: 10.1016/0028-3932(90)90004-8.

[31] A R Mayes, C L Isaac, J S Holdstock, N M Hunkin, D Montaldi, J J Downes, C Macdonald, E Cezayirli, and J N Roberts. Memory for single items, word pairs, and temporal order of different kinds in a patient with selective hippocampal lesions. *Cognitive neuropsychology*, 18(2):97–123, 2001. doi: 10.1080/02643290125897.

[32] N Fortin, K Agster, and H Eichenbaum. Critical role of the hippocampus in memory for sequences of events. *Nature Neuroscience*, 5(5):458–62, 2002. doi: 10.1038/nn834.

[33] L Devito and H Eichenbaum. Memory for the order of events in specific sequences: contributions of the hippocampus and medial prefrontal cortex. *The Journal of Neuroscience*, 31(9):3169–75, 2011. doi: 10.1523/jneurosci.4202-10.2011.

[34] Peter Dayan, S Kakade, and P Montague. Learning and selective attention. *Nature neuroscience*, 3 Suppl VN(Supp):1218–1223, 2000. doi: 10.1038/81504.

[35] Nelson Rebola, Mario Carta, and Christophe Mulle. Operation and plasticity of hippocampal CA3 circuits: Implications for memory encoding. *Nature Reviews Neuroscience*, 18(4):209–221, 2017. doi: 10.1038/nrn.2017.10.

[36] Pierre Perruchet. What Mechanisms Underlie Implicit Statistical Learning? Transitional Probabilities Versus Chunks in Language Learning. *Topics in Cognitive Science*, pages 1–16, 2018. doi: 10.1111/tops.12403.

[37] Andrew E. Papale and Bryan M. Hooks. Circuit Changes in Motor Cortex During Motor Skill Learning. *Neuroscience*, 368:283–297, 2018. doi: 10.1016/j.neuroscience.2017.09.010.

[38] J. Doyon, E. Gabitov, S. Vahdat, O. Lungu, and A. Boutin. Current issues related to motor sequence learning in humans. *Current Opinion in Behavioral Sciences*, 20:89–97, 2018. doi: 10.1016/j.cobeha.2017.11.012.

[39] John W. Krakauer and Reza Shadmehr. Consolidation of motor memory. *Trends in Neurosciences*, 29(1): 58–64, 2006. doi: 10.1016/j.tins.2005.10.003.

[40] M Page, N Cumming, D Norris, A McNeil, and G Hitch. Repetition-spacing and item-overlap effects in the Hebb repetition task. *Journal of Memory and Language*, 69(4):506–526, 2013. doi: 10.1016/j.jml.2013.07.001.

[41] Ahna R Girshick, Michael S Landy, and Eero P Simoncelli. Cardinal rules: visual orientation perception reflects knowledge of environmental statistics. *Nature Neuroscience*, 14(7):926–932, 2011. doi: 10.1038/nn.2831.

[42] Marijke Welvaert and Yves Rosseel. On the definition of signal-to-noise ratio and contrast-to-noise ratio for fMRI data. *PLoS ONE*, 8(11), 2013. doi: 10.1371/journal.pone.0077089.

[43] M Page and D Norris. A model linking immediate serial recall, the Hebb repetition effect and the learning of phonological word forms. *Philosophical transactions of the Royal Society of London. Series B, Biological sciences*, 364(1536):3737–53, 2009. doi: 10.1098/rstb.2009.0173.
